# Is the Prosodic Structure of Texts Reflected in Silent Reading? An Eye-Tracking Corpus Analysis

**DOI:** 10.3390/jemr18030024

**Published:** 2025-06-18

**Authors:** Marijan Palmović, Kristina Cergol

**Affiliations:** 1 Laboratory for Psycholinguistic Research, Department of Speech & Language Pathology, University of Zagreb, 10000 Zagreb, Croatia; 2 Laboratory for Psycholinguistic Research, Faculty of Teacher Education, University of Zagreb, 10000 Zagreb, Croatia

**Keywords:** implicit prosody, silent reading, reading corpus, implicit prosody hypothesis, stress-timed rhythm, syllable-timed rhythm

## Abstract

The aim of this study was to test the Implicit Prosody Hypothesis using a reading corpus, i.e., a text without experimental manipulation labelled with eye-tracking parameters. For this purpose, a bilingual Croatian–English reading corpus was analysed. In prosodic terms, Croatian and English are at the opposite ends of the spectrum: English is considered a time-framed language, while Croatian is a syllable-framed language. This difference served as a kind of experimental control in this study on natural reading. The results show that readers’ eyes lingered more on stressed syllables than on the arrangement of stressed and unstressed syllables for both languages. This is especially pronounced for English, a language with greater differences in the duration of stressed and unstressed syllables. This study provides indirect evidence in favour of the Implicit Prosody Hypothesis, i.e., the idea that readers are guided by their inner voice with its suprasegmental features when reading silently. The differences between the languages can be traced back to the typological differences in stress in English and Croatian.

## 1. Introduction

In psycholinguistics, experiments are traditionally the standard method for testing hypotheses. A typical experiment consists of visually or auditorily presented stimuli and a measuring device that records the values of the dependent variable in response to the experimental manipulation of the stimuli. A common criticism of this type of psycholinguistic study is its low ecological validity, although simplifying the test situation and only manipulating one variable are emphasised as clear advantages of the experimental method in the study of automatic language processing. On the other hand, the emerging method of collecting a reading corpus enables analyses based on a large amount of data and various word features. It also minimises the demands of the task, reducing the likelihood of strategic biases that can influence the results [1,2].

After all, who really uses language by sitting in front of a computer screen while sequences of letters flash before their eyes? The measurement of eye movements is no exception. Strict experimental control of the stimuli is often even more necessary than, for example, when measuring reaction time, as many confounding variables associated with oculomotor or motor processes can influence the recorded values. This, in turn, makes the need for experimental control even greater, which makes such experiments appear even less natural.

In recent years, eye-tracking methodology has begun to be used in a different way: participants are asked to read texts that are not experimentally manipulated. These texts are often newspaper articles, short stories or popular science texts. They usually comprise around ten pages, but sometimes more, and are read by around one hundred participants. This method of data collection has been given a cumbersome name—natural reading—and the data are organised into reading corpora, another somewhat unwieldy term. Reading corpora include data on texts together with data from eye-tracking devices. Today, there are several such corpora, including the GECO [1], with extensions for English–Chinese bilingual speakers [3]; the Copenhagen Corpus of Eye Tracking Recordings from Natural Reading of Danish Texts [4]; the German naturalistic eye-tracking-while-reading corpus [5]; the Provo Corpus [6], a corpus containing predictability norms (i.e., statistical measures that indicate how predictable a word in a paragraph is); and the TURead, a Turkish corpus comprising 192 short texts [7]. In recent years, the corpus method in eye-tracking has established itself as one that enables the analysis of language processing in naturally occurring language use.

**Figure 1:**
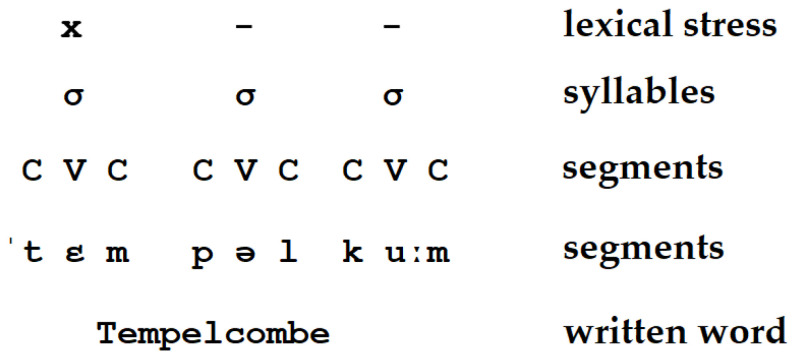
A simplified view of the hierarchical nature of phonological representation, including lexical stress.

The idea that prosody is in some way reflected in silent reading is not new; in fact, it is more than a century old [8]. Huey introduced the term “inner voice”, meaning that a reader cannot escape the silent pronunciation of words with their prosodic patterns. The reader’s brain creates this “voice”, which contains lexical stress, in addition to rhythm and intonation. In modern terminology, this boils down to the fact that phonological representations (or codes) are activated during silent reading. According to modern phonological theories, these representations are hierarchical in nature [9,10,11,12,13] and contain prosodic information, as shown in Figure 1, which illustrates the levels of phonological representation for the word Tempelcombe, a train station mentioned in a story selected from the Croatian–English corpus.

The empirical evidence for the hierarchical organisation of phonological information comes from language acquisition studies [14], which suggest that children follow a structured approach to the acquisition of phonological elements, with each unit playing a specific role in the overall hierarchy of phonological development. Further evidence comes from second-language acquisition studies (for a review, see [15]), which show how stress, intonation, and rhythm influence the intelligibility and comprehension of the second language.

The visual recognition of words includes the identification of letters and letter patterns, access to the meaning of words and their pronunciation, and their integration into larger structures [16]. Beyond individual words, the prosodic patterns shape the parsing of sentences and help to integrate the information at the discourse level. Prosodic patterns provide cues that help the listener to anticipate the organisation of a sentence or reduce its ambiguity and are of central importance for sentence comprehension [17]. In this way, the inner voice helps the reader to “hear” where the emphasis lies or which words belong together. Early empirical evidence for this mapping from orthography to phonology makes use of English orthography, with the resulting abundance of homophonous words: for example, the word hare is often incorrectly mapped to a human body part (hair) in a categorisation task, and pseudowords (e.g., sute) are classified as belonging to a proposed category, namely “an article of clothing” [18]. In similar eye-tracking studies, the ability to control parafoveally presented information was utilised in gaze-contingent experiments. It was found that homophones facilitated the word recognition process (as an example, fixations on the target word beach were shorter when the parafoveal mask featured the pseudoword beech and not the word bench) [19]. These findings clearly point to the activation of phonetic codes or the “inner voice” in reading. The Implicit Prosody Hypothesis [20] goes a step further; it claims that a speaker follows the prosodic structure of the text when reading silently. As pointed out by Clifton [21], it is the prosodic contour that guides a reader in comprehending the written text by placing pauses and breaks, helping to identify the groupings of words or following the information structure of a sentence. Finally, the prosodic structure of a written text influences eye movements during reading by guiding the eye to where in the text and for how long it should linger.

The role of prosody in resolving ambiguity or recognising constituent structure was recognised even before Fodor’s formulation of the Implicit Prosody Hypothesis (for a review, see [22]). Various techniques were employed prior to the introduction of eye-tracking. For example, the role of prosody in reading comprehension was studied using the concept of “forced” vocalisation, which interfered with the assumed subvocalisation that accompanies silent reading [23]. Forced vocalisation (participants were asked to say “Coca-Cola, Coca-Cola…” while reading or listening) impaired reading comprehension but not listening comprehension. Another way of investigating the role of prosody was to conduct experiments in which participants were presented with clicks together with stimulus sentences. The reaction times to these clicks “migrated” towards the constituent boundaries [24], i.e., the prosodic structure of the sentences influenced the perception of the timing of the clicks.

More recently, empirical evidence for the Implicit Prosody Hypothesis has been sought by examining neuromuscular activity in the head and neck during silent reading [25], measuring event-related potentials associated with listener expectations based on prosody [26], or using eye-tracking experiments with stressed English homophones belonging to different parts of speech (ADDress vs. addRESS, PREsent vs. preSENT [27]). Readers’ difficulty in processing stress-incongruent words was reflected in the go-pass time (total fixation time from first entry into the region of interest (target word) to its exit to the right), fixation probabilities, regression probabilities, and second-pass time, rather than first-pass time. This suggests that readers do not have difficulty with initial word processing, but that stress slows them down as they continue to read the text. Similarly, the effects observed on the dwell times (i.e., sums of fixation durations in an area of interest), and not on the first-pass fixation variable, led to the conclusion that the phonological code is activated during silent reading, but this code is incomplete [28].

The role of prosody in syntactically ambiguous contexts was analysed in a syntactic reference resolution study [29]. The German phrase “nicht mehr” was used either as a phrase in the sense of “no longer” with the stress on the first word (e.g., Der Polizist sagte, dass man NICHT mehr ermittleln kann, wer der Taater war; The policeman said that one couldn’t determine anymore who the culprit was) or as two words, with the second (mehr) being an adverb that complements the verb (…dass man nicht MEHR ermitteln kann, als die Tatzeit; …that one couldn’t determine more than the date of the crime). The results showed that for the three words in the ambiguous region (nicht mehr + verb), there was a significant increase in first-pass regressions and the probability of re-reading. This was taken as evidence of the processing difficulties that occur when readers encounter a conflict between the prosodic contour and the syntactic structure. An eye-tracking study employing a picture matching task was employed in [30]. In two experiments, adjective–noun combinations were used either as phrases (e.g., yellow jacket) or as compounds (e.g., hot dog or white house), with corresponding differences in the acoustic cues. The results corroborated the claim that listeners effectively use prosody for compounds they know the meaning of but rely on lexical information when faced with novel combinations. In an experiment with a visual world paradigm, it was found that contrasting pitch accents, especially with adjectives, help listeners to identify referents more quickly. For example, when instructed to “click on the RED scissors”, the contrasting accent led participants to anticipate the target object among the other objects in the scene [31].

The approach taken by Bishop [32] attempted to relate explicit and implicit prosody by exploring individual differences. The author found that individual differences in explicit prosody (participants had to read English text passages aloud) could be predicted by differences in working memory capacity and that individual differences in explicit prosodic phrasing predicted individual differences in implicit prosodic phrasing. The second step was achieved by self-assessments after the silent reading of text passages. Finally, Beck and Konieczny [33] investigated how rhythmic patterns influenced eye movements during the silent reading of poetry. They found that metrical anomalies in poems led to interruptions in reading, which were reflected in longer fixation and reading times. Metrical anomalies had less influence on the silent reading of a prose text.

To our knowledge, corpus-based eye-tracking studies have not looked for evidence that would confirm the Implicit Prosody Hypothesis. In a study by Tong et al. [34], paragraph-reading tasks with pictures and multiple-choice questions were used in an eye-tracking experiment that investigated the relationship between prosody and reading comprehension in bilingual Chinese and English children. The study established the mediating role of syntactic awareness (i.e., the ability to recognise syntactic structures and understand how these structures fit together), but also the dependence of this mediation on language-specific prosody. The idea that prosody influences language comprehension processes is further developed here not only by comparing two languages that show typological differences in prosody (such as English and Chinese), but also by comparing English with Croatian, a language in which the influence of prosody on language comprehension is expectedly lower than in English, leading to the prediction of a greater influence of prosody in the second language, i.e., English. This is in contrast to the results in [34] and other eye-tracking studies focussing on the role of prosody in language comprehension [35,36]. Other psycholinguistic studies [37] also emphasise the importance of transfer and cognitive mapping of prosody from the first to the second language. In this study, however, the focus is on the specific role of prosody in each language and not on the processes involved in language learning, such as the transfer mentioned above. Instead of seeking evidence for the Implicit Prosody Hypothesis using psycholinguistic experiments, data were collected by using a reading corpus, as the latter is a more natural (ecologically valid) method of obtaining reading data and, therefore, better reflects whether speakers follow the stress structure of a multi-page text. Reading the entire text instead of individual sentences makes a difference when measuring eye movements. In continuous texts, eye movements are largely controlled by the lexical properties of the words, such as frequency or predictability. Context tends to affect processing speed by making it easier to anticipate the next words. This speeds up reading and affects eye-tracking metrics such as fixation duration or saccade length [38]. However, a kind of experimental control was maintained by exploiting the typological differences between Croatian and English in terms of stress patterns. English and Croatian differ in their prosodic structure on many levels. In English, one speaks of phonemic stress, which can have the function of distinguishing between words (INsight vs. inCITE) or be used to derive nouns (preSENT vs. PREsent). Standard Croatian has four pitch accents: short and long rising and falling accents. Falling accents occur in monosyllabic words, while both rising and falling accents can occur in polysyllabic words, with rising accents never occurring on the last syllable. If a clitic precedes a monosyllabic noun, the accent jumps from the noun to the clitic and becomes a rising accent. If a case marker is added to the noun and a syllable is added, the falling accent becomes a rising accent (e.g., grâd city.NOM.SG (long, falling accent), ȕ͜ grad to the city.ACC.SG (short falling accent), but u grádu in the city.LOC.SG (long rising accent)). So, the Croatian accent is predictable to a certain extent.

More importantly, English is said to have a time-framed rhythm, while Croatian is considered a language with a syllable-framed rhythm [39]. In languages with a time-framed rhythm, the duration between the stressed syllables is relatively constant; this leads to variable lengths of the unstressed syllables. In languages with a syllable-framed rhythm, syllables tend to have a similar duration [40]. In other words, when an English speaker starts to speak faster, they tend to “swallow” the unstressed syllables. On the other hand, when a Croatian speaker speaks faster, they shorten all syllables proportionally; it is sometimes said that languages with syllable-framed rhythm sound like a machine gun.

The relatively longer duration of stressed syllables in English thus makes them more conspicuous. This allows for the direct hypothesis that stressed syllables attract more attention in English than in Croatian. Therefore, longer fixation durations (dwell times) and more fixations are expected for stressed syllables, but more so in English. If this is the case, it would confirm the Implicit Prosody Hypothesis by showing that eye movements during silent reading are more dependent on stress in languages with greater differences in the prominence of stressed and unstressed syllables. The bilingual nature of the corpus thus serves as a kind of experimental control in a method that inherently lacks one.

## 2. Materials and Methods

*Materials.* The text used for the collection of the eye-tracking data was the short story “Storyteller” by Hector Munro Saki [41]. A translation of the story was used for the data collection in Croatian [42]. The English text contained 1962 words and 2696 syllables, of which 1835 were stressed. The Croatian translation consisted of 1746 words with 3614 syllables, of which 1266 were stressed. On average, English words were 1.4 syllables long, while Croatian words were longer, averaging 2.1 syllables. This difference is due to the typological differences between the two languages. Croatian case endings often add a syllable. For example, the English phrase “to the boy” corresponds to the Croatian dative “dječak-u”, which means that three monosyllabic English words correspond to a single three-syllable Croatian word.

Finally, the English text contained 8933 letters, while the Croatian translation had 8588 letters. English words were, on average, 4.6 letters long, while Croatian words were, on average, 5 letters long. This similarity in length despite the difference in the number of syllables can be explained by the difference in orthographic depth in English and Croatian, which is expressed by the ratio of graphemes to phonemes. More precisely, this ratio is expressed as the number of grapheme–phoneme correspondences divided by the number of graphemes (GPC/g, [43]). In Croatian, this ratio is 1.1:1; in English, it is 2.4:1. In other words, a similar number of letters in Croatian and English texts encode fewer phonemes in English.

Participants. The participants were university students, who were native speakers of Croatian with English proficiency level B2 or higher. The participants’ English-language skills were not tested for this study. Instead, their score on a nationwide test was considered sufficient as they were all students who used English daily in class. A total of 51 participants took part in the study, but only data for 45 (F = 42, M = 3) were analysed in this study; the included data met the high standard of accuracy required for a reading task. Each participant took part in two measurement sessions. Half of the participants (*N* = 22) read the text in English on the first visit; the other half read it in Croatian *(N* = 23). The interval between the two sessions was at least two weeks.

Procedure. Eye movements were recorded with the SR Research EyeLink Portable Duo eye-tracker, Ottawa, Canada at a sampling rate of 1 kHz. A chin rest was used to stabilise the participants’ heads. Participants sat 60 cm from the screen, read the instructions, and then underwent 9-point calibration and validation. This process was repeated until an accuracy of <0.35° was achieved. Reading was self-paced; participants moved to the next page by pressing the space bar, with drift correction performed before each page to ensure calibration accuracy during the multi-page measurement (if drift was high, calibration was performed again during reading). After completing the measurement, the participants answered three questions to assess text comprehension. The questions were related to details that the participants were supposed to remember (e.g., how many medals Bertha, the girl in the story, wore on her dress), as well as the general message of the story (i.e., it often does not pay to be good).

Before reading “Storyteller”, the participants read a short fable by Aesop as an exercise. The corpus, in both Croatian and English, consisted of 11 pages of text with 12 lines each, written in Courier New font, size 16.

Analysis. Once the measurement was completed, the data were exported based on areas of interest defined around stressed and unstressed syllables. These data were pre-processed for statistical analysis using R 4.4.3 [44] and the tidyverse package [45]. The statistical analysis was also performed in R using the BayesFactor and brms packages [46,47]. For the statistical analysis, a Bayesian logistic regression with stress as a categorical two-level variable (“stress” and “no_stress”) and continuous predictor variables (dwell time, duration of first-pass fixation, number of fixations, and number of regressions) was used, with participants serving as a random predictor. The aim of this model was to show whether the eye-tracking pattern alone could predict whether a syllable was stressed or not. Variables that reflect both early and late processes of reading comprehension were included in the analysis. First-fixation duration (FFD) is assumed to reflect the orthographic, phonological, morphological, and lexical processes involved in lexical activation [48]. The dwell time (i.e., the time from entry to exit from an area of interest) is thought to reflect the “postlexical” processes, including the integration of a word into the sentence context. Dwell time is generally regarded as an indicator of interest in an object (word) or its higher information content. This means that if the reader’s attention is more focussed on stressed syllables, it is these stressed syllables that provide most of the information that the reader needs to understand the sentence. Regressions are generally thought to be related to difficulties in extracting meaning, a kind of double-checking of the previously fixated word [49]. The fixation count variable is related to a higher engagement of attention, i.e., the greater the number of fixations in an area of interest, the greater its interest value [50]. The fixation and regression count variables were considered in the analysis as they are assumed to be less colinear with the duration variables and could thus be informative for the model.

This study received ethics approval from the ethics committee of the first author’s faculty, registry number 251-74/22-02/7-2.

## 3. Results

**Figure 2:**
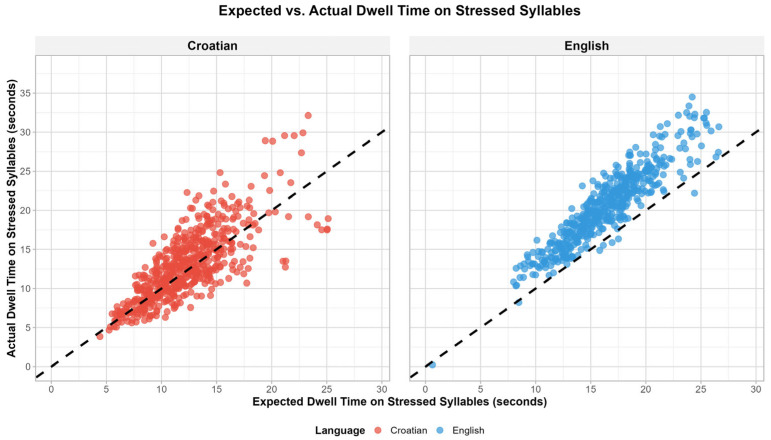
Expected vs. actual dwell time on stressed syllables. The points represent the participants.

The results show that the participants spent more time fixating on the stressed syllables than would be expected based on their percentages alone (see Figure 2). Figure 2 shows the time spent in the study per participant and language. The figure clearly shows that the same participants paid more attention to stressed syllables when reading in English than when reading in Croatian. The overall lower dwell times (their sums per trial per participant) in Croatian than in English indicate that the participants have a better command of Croatian, their mother tongue.

**Figure 3:**
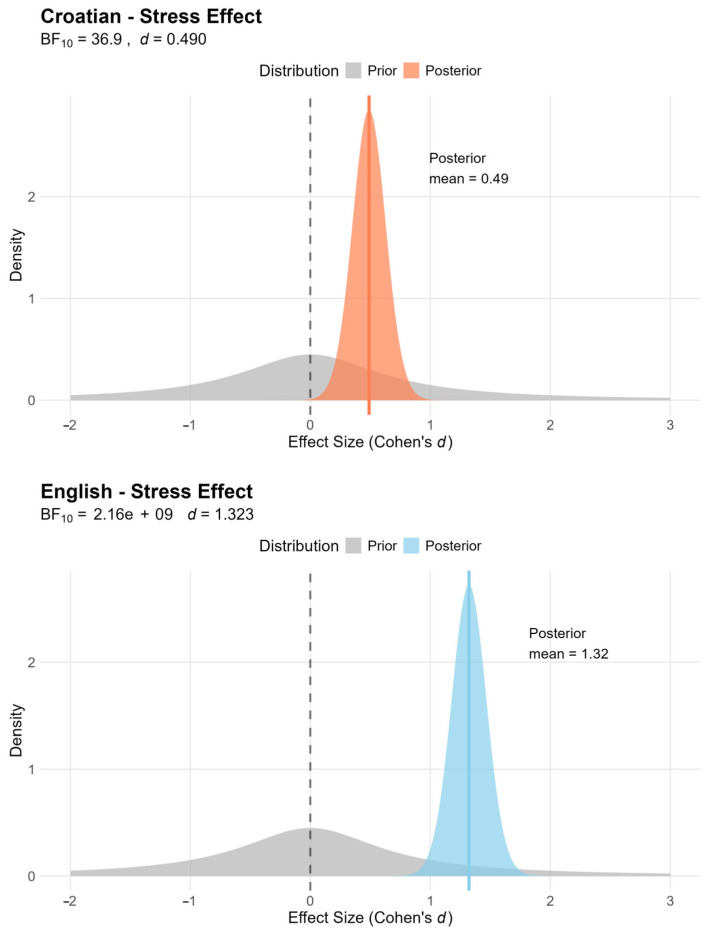
The effect of stressed syllables in English and Croatian on the dwell time variable.

To test the hypothesis that readers follow the prosody of the text even when reading silently, an analysis based on a Bayesian *t*-test was performed for the dwell time variable, where the prior was calculated based on the expected dwell time on the stressed syllables. This was based on the prosodic structure of the text, i.e., the sum of all dwell times on stressed and unstressed syllables per participant was multiplied by the proportion of stressed syllables in the texts. These values are different for the two languages: in Croatian, this proportion is 0.37, and in English, 0.68. The calculation of this value per participant provides the mean value and the standard deviation of the sample, which are required for the calculation of the priors. The results of the Bayesian *t*-test are shown in Table 1. The results show that readers spent 32% more time than expected on stressed syllables in English and 9% more time than expected in Croatian. The t-values are very high for English and moderate for Croatian. The Bayes factor is extremely high in English and high in Croatian, indicating that our hypothesis is highly probable, i.e., it is likely that fixations are directed more towards the stressed syllables when reading in a language in which stressed syllables are more conspicuous. Finally, Cohen’s d shows a large effect for English and a moderate effect for Croatian in the same group of participants. The effect of stress in English and Croatian is shown in Figure 3.

These results can be interpreted as a tendency among readers to focus on the most salient and informative parts of words during silent reading, namely the stressed syllables. These findings are consistent with the idea of the predictive role of prosody in sentence comprehension, as dwell time reflects the postlexical processes of integrating a word into sentence meaning [48,49,50].

However, the eye-tracking method makes it possible to record various processes involved in sentence comprehension. A measure that reflects rapid word recognition processes is also relevant to the “inner voice”, as some studies suggest [17,50]. The predictive role of prosody presupposes that the reader progresses quickly through the text due to easier word recognition. This would be captured in the “early” eye movement variables, such as FFD. The analysis, which allows for conclusions based on the different interpretations of various eye-tracking variables, provides a sharper view of the comprehension process and utilises the availability of the multitude of variables provided by the eye-tracker. Therefore, we included a logistic regression model with the categorical outcome variable “stress”, several predictor variables provided by the eye-tracker, and the participants as a random predictor in order to obtain a more accurate picture of the processes influencing the increased fixation on the stressed syllables during silent reading. However, prior to the analysis, the distributional properties of the relevant predictors and the assumptions for the logistic regression were tested. The dataset comprised 309,992 observations from 45 participants, with 157,779 observations (51%) retained after the removal of missing values (e.g., skipped areas of interest). The FFD variable (M = 203.40 ms, SD = 85.72) exhibited positive skewness (skewness = 1.94), necessitating a log transformation. The number of fixations (M = 1.29, SD = 0.69) was retained as a count variable, with 78% of observations showing a single fixation in an area of interest. Regrettably, dwell time was excluded from the final model due to high multicollinearity with the number of fixations (r = 0.79, *p* < 0.001), which could lead to unstable parameter estimates. Finally, regressions in the areas of interest were excluded as they showed no significant relationship with syllable stress (χ^2^ = 0.003, *p* = 0.958) and showed extreme class imbalance (79% zeros). The transformations of the variables were performed as follows: FFD was log-transformed (log(x + 1)) to counteract positive skewness. All continuous predictors were then standardised (z-scored) to facilitate the interpretation of the coefficients. The correlation between the log-transformed FFD and the normalised fixation count variable was 0.052. The formula used for the logistic regression was

stress ~ FFD+FIXATION_COUNT+(1|PARTICIPANT)).

The logistic regression model predicts whether a syllable is stressed or not solely based on the two parameters of eye movement. Both measures are “local”, i.e., they are based on the areas of interest. The first-fixation duration measures only the first fixation in an area of interest (stressed or unstressed syllables), while the number of fixations simply counts the fixations within the areas of interest.

The analysis revealed differences between the two languages indicative of the different roles of stress in sentence comprehension in English and Croatian. When reading the English text, participants showed effects both for the duration of the first fixation (OR = 1.151, 95% CrI: [1.086, 1.221]) and for the number of fixations (OR = 1.314, 95% CrI: [1.208, 1.427]). In contrast, when reading in Croatian, the effect was only found for the number of fixations (OR = 1.297, 95% CrI: [1.143, 1.469]) and not for the duration of the first fixation (95% CrI: [−0.007, 0.109]). These results suggest that in English, stress plays a greater role in both word recognition and “postlexical” processes. When reading Croatian texts, the same participants used the stress information mainly in the “postlexical” phase. The results are shown in Table 2.

**Figure 4:**
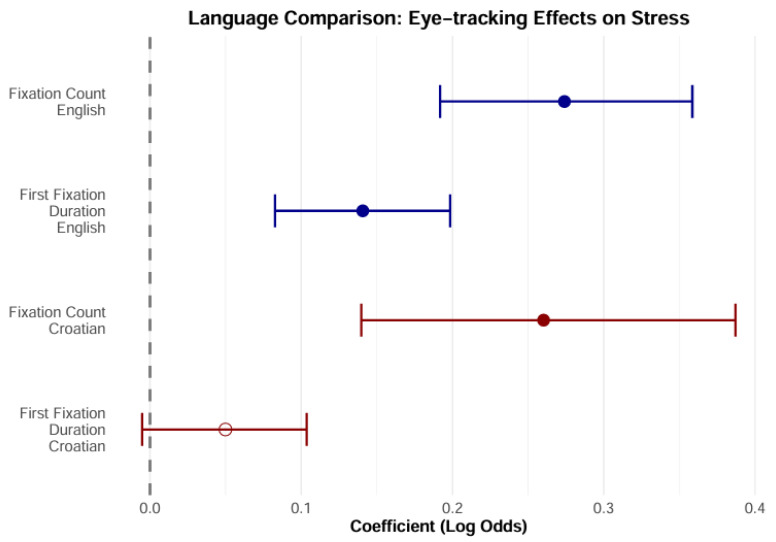
Odds ratio for English and Croatian.

The negative intercept indicates that syllables are more likely to be unstressed than stressed for the average values of all predictors, and more so in Croatian than in English. This simply reflects the linguistic (prosodic) structures of both languages—there are more unstressed syllables in both languages, and more so in Croatian. The results are shown graphically in Figure 4.

To summarise, these results indicate that, in English, the probability of the first fixation landing on the stressed syllable increases by 15.1% for each standard deviation that the log(FFD) increases, and the probability of fixations landing on the stressed syllable increases by 13.1% for each additional fixation. In Croatian, these odds are different: the chance of the first fixation landing on the stressed syllable is only 5.1% higher per standard deviation (log(FFD)), but the chance for each additional fixation is 29.8% higher. In this model, the duration of the first fixation and the number of fixations proved to be good predictors of syllable stress, at least in English. The high predictive power of the first-fixation duration indicates that prosodic information is available early in the process of lexical processing. As mentioned in the Introduction, stress in English can have a function in distinguishing between words. The postlexical processes reflected in the fixation counts may indicate that prosody contributes to the integration of the word into the sentence context in both languages. This result is thus consistent with the Implicit Prosody Hypothesis, i.e., the idea that readers impose the prosodic contour on the text and that this contour contributes to the control of eye movements during reading. The differences found between English and Croatian point to the typological differences in prosody between the two languages.

Individual differences were analysed to obtain a clearer picture of the role of stress in silent reading. Can the differences between the languages be attributed to the differences in prosodic structure or to individual differences between the participants only? Individual correlation coefficients between the predictors of eye-tracking and the identification of stress were calculated for each participant. Interestingly, analyses at the group level showed that, compared with stress in Croatian, stress in English was better predicted by the first-fixation duration variable, while this pattern varied greatly at the individual level. The strength of the individual correlations for first-fixation duration did not differ significantly between English (M = 0.037, SD = 0.040) and Croatian (M = 0.049, SD = 0.035), t (17.71) = 0.93, *p* = 0.365. The individual effects of the number of fixations (fixation counts) were also comparable between the languages (*p* = 0.128). These results indicate considerable individual variability within the two language groups, suggesting that processing strategies may be influenced by more than just language. While there are tendencies in each language that can be linked to typological differences in prosodic structure, individual readers may develop unique approaches to the use of stress information that transcend language-specific patterns. This emphasises the importance of considering both language-level patterns and individual processing strategies when attempting to understand cross-linguistic differences in prosodic processing.

## 4. Discussion

There is a wealth of evidence in favour of the Implicit Prosody Hypothesis. In research using the eye-tracking method, this evidence is based on participants reading individual sentences or performing a picture-viewing task while listening to the stimulus texts through their headphones. To our knowledge, there are no studies based on reading corpora that examine implicit prosody or inner speech. In this study, a bilingual corpus was used to test the Implicit Prosody Hypothesis by utilising the typological differences in prosody between English and Croatian.

The logic of this study was simple: it tested the Implicit Prosody Hypothesis by showing that (1) readers’ eyes lingered more on stressed syllables than would have been expected based on prosodic structure alone, and (2) that this effect was greater in a language where stressed syllables are more prominent in terms of duration than in a language where all syllables are approximately the same length. The difference in prosodic typology thus represents the experimental control in a natural reading study (where, by definition, there is no control). A simple comparison between what would be expected from the prosodic structure alone and the eye-tracking data clearly points to this conclusion. In other words, the excess time that readers spend on stressed syllables compared to the expected time can be attributed to the “inner voice” that directs their gaze.

Using the multitude of variables provided by the eye-tracker to predict whether syllables are stressed or not is more complex. Optimally, this analysis could provide insights into the phases of sentence processing based on the different interpretations of the individual eye-tracking variables. It is hypothesised that the duration of the first fixation in a region of interest (FFD) reflects the processes involved in word recognition [48], while the total time spent in a region of interest (dwell time) reflects the processes related to postlexical processes [28]. It is due to collinearity problems that the dwell time variable was not included in the logistic regression model but only compared between Croatian and English. Instead, the number of fixations, a variable associated with higher attentional engagement, was included in the model. Both FFD and the number of fixations proved to be good predictors of stressed syllables in English. Since stressed syllables last phonetically longer in English (i.e., in open speech) [39,40], the fact that readers’ eyes linger more on stressed than unstressed syllables during silent reading points to the Implicit Prosody Hypothesis. In other words, stress plays a role in both word recognition and in predicting sentence structure. In Croatian, this phonetic difference in the duration of stressed and unstressed syllables is smaller. Stress was not found to influence lexical processes (as observed in FFD). On the other hand, the results for the fixation variable are similar to those obtained in English. The difference between the results in English and Croatian can be seen as further evidence in favour of the Implicit Prosody Hypothesis, as there were more and longer fixations on stressed syllables in the language with a time-framed rhythm, even in a study with unbalanced bilinguals. Stress as a linguistic cue was found to be different in English and Croatian for the same group of participants, and this difference can be explained by the typological differences between the two languages.

However, these results differ from those obtained in eye-tracking studies on the predictive role of prosody in bilinguals [35,36]. In these studies, prosody was generally found to play a lesser role in predicting sentence meaning in the second language. Based on these studies, one would have anticipated a lesser predictive role of dwell time or the number of fixations in English. The difference lies in the methodology: these results were all obtained in visual world experiments with contrastive stress (e.g., “We ate Angela’s CAKE, but not her ice cream”) and anticipatory gaze as the dependent variable (how much time in advance the participants focused their gaze on the cake in the picture). In a recent study on contrastive stress in first and second languages [51], the evidence for the predictive use of prosodic cues was less clear for second-language learners than for native speakers. This was neither related to working memory (which was controlled for in this study) nor, interestingly, to fluency in the second language. These results were explained within the framework of the “complex mapping account” [52], which states that second-language learners can integrate information from different domains (syntactic, lexical, prosodic) but only if this integration is easy.

In short, at the group level, it seems that the duration of the first fixation predicts stress better in English than in Croatian. Fixation counts are similar in both languages. However, when looking at individual participants, this pattern is not consistent. The strength of the relationship between first-fixation duration and stress does not really differ significantly between English and Croatian. The same applies to fixation counts. This means that there are large differences between individual readers, i.e., some readers processed stress similarly when reading the English text as they did when reading the Croatian text, while they differed from other readers of the English text. In other words, while languages have their own typical patterns for processing stress, individual readers seem to develop their own personal strategies that sometimes go beyond what is typical for their language. Therefore, when we study how people process rhythm and stress in different languages, we need to consider both language-specific patterns and individual reading strategies.

However, some limitations of this study must be considered. (1) Prosody is only one of many factors that guide the eyes when reading. There are many other linguistic features that attract the eye when reading; it is known that some words are skipped more often than others. There are also some non-linguistic features, purely visual ones, such as the effect of optimal viewing position, according to which the centre of a word has a greater chance of being fixated on than its beginning or end. In both English and Croatian, the stress is mainly on the first syllable, but Croatian suffixes also contain morphological information necessary for the integration of a word into the textual context. In fact, English and Croatian are morphologically different; English is usually categorised as an analytic language, while Croatian is a fusional language. All these features cannot possibly be considered in a reading corpus if the analysis is based on the areas of interest created around a single linguistic feature. (2) Participants were selected based on their score in a nationwide English test and the fact that they use English daily. With a group of balanced Croatian–English bilinguals, the results might have been different. Unfortunately, such a group of participants was not available at the time of this study, and English was selected based on typological considerations. The convenience sample used in this study was also unbalanced regarding the gender of the participants. Although there is no reason to expect different results, future studies should use a more balanced sample in terms of age, gender, or education. (3) Some Croatian dialects differ in the position of stress. Although the story of Saki was written in standard Croatian and the stress of the syllables was marked according to the Croatian standard, some readers may have read the text in their dialectal inner voice. This could have influenced the results and made the Croatian stress seem less important than it really is. The dialectal origin of the Croatian participants will therefore be included as information in the development of a new Croatian reading corpus.

## Figures and Tables

**Table 1:** Results of Bayesian *t*-test on dwell time.

Language	Mean Stress Ratio	T-Statistics	BF	Cohen’s D
Croatian	1.09	3.52	36.87	0.49
English	1.32	9.07	2.16 × 10^9^	1.32

**Table 2:** Bayesian mixed-effects logistic regression results.

Parameter	β (SE)	95% CI	OR [95% CI]	*p* (β > 0)	R-Hat
English					
Intercept	−0.468 (0.065)	[−0.594, −0.341]	0.626 [0.552, 0.711]	-	1.00
log(FFD)	0.141 (0.030)	[0.082, 0.199]	1.151 [1.086, 1.221]	1.000	1.00
Fixation Count	0.273 (0.042)	[0.189, 0.355]	1.315 [1.208, 1.427]	1.000	1.00
Croatian					
Intercept	−0.832 (0.087)	[−1.002, −0.661]	0.436 [0.367, 0.516]	-	1.00
log(FFD)	0.050 (0.030)	[−0.007, 0.109]	1.051 [0.993, 1.115]	0.956	1.00
Fixation Count	0.261 (0.064)	[0.134, 0.385]	1.297 [1.143, 1.469]	1.000	1.00

## Data Availability

A bilingual Croatian–English reading corpus was compiled for this study. The authors intend to make it publicly available. At the time of submission, the data are available upon request.

## Abbreviations

The following abbreviations are used in this manuscript:

NOM	Nominative
ACC	Accusative
LOC	Locative
SG	Singular
GPC	Grapheme–phoneme correspondence
BF	Bayes factor
CI	Credible interval
OR	Odds ratio
FFD	First-fixation duration
DT	Dwell time
Fix.Count	Fixation count
Regress. Count	Regression count
